# Cancer in Santiago Island, Cape Verde: data from the Hospital Agostinho Neto Cancer Registry (2017–2018)

**DOI:** 10.3332/ecancer.2019.995

**Published:** 2019-12-18

**Authors:** Elizeu Teixeira Silva, Hirondina Borges Spencer, Victor Costa, Ana Filipa Gonçalves, Clara Castro, Maria José Bento, Carla Barbosa, Lúcio Lara Santos

**Affiliations:** 1 Hospital Central Agostinho Neto, Rua Borjona de Freitas, Plateau 112, Praia, Cape Verde; 2 Departamento de Epidemiologia, e Grupo de Investigação de Epidemiologia do Cancro, Instituto Português de Oncologia, 4200-072 Porto, Portugal; 3 Coordenação das Doenças Oncológicas de Cabo Verde, Rua Borjona de Freitas, Plateau 112, Praia, Cape Verde; 4 Departamento de Cirurgia e Grupo de investigação de Patologia e Terapêutica experimental, Instituto Português de Oncologia, 4200-072 Porto, Portugal

**Keywords:** Santiago Island, Cape Verde, Hospital Agostinho Neto, cancer registry

## Abstract

This report describes the cancer cases that occurred between 2017 and 2018 in Santiago Island, Cape Verde, according to the Hospital Agostinho Neto Cancer Registry. The five most common cancers were prostate, breast, stomach, cervix and oesophageal in order of frequency. There are no national data. Therefore, it is essential to create the conditions for the establishment of Cape Verde’s population-based cancer registry as quickly as possible.

## Background

Santiago is the largest island of Cape Verde; it is the most important agricultural centre and home to half the nation’s population. Santiago is home to the nation’s capital city of Praia [1].

Life expectancy at birth according to data from the World Bank (2017) is 73 years (female 75 years, male 71 years). The mortality rate was 98/1,000 for female and 144/1,000 for male adults [2]. Cape Verde experiences a high burden of non-communicable diseases, the most significant being cardiovascular-related diseases, diabetes and cancer. According to the Institute for Health Metrics and Evaluation, in 2017, cancer was the second cause of death in Cape Verde (17.9%) [3].

In Santiago Island, the Hospital Agostinho Neto (HAN) has a unit for the diagnosis and treatment of cancer which includes surgery and chemotherapy and treats most of the cancer patients because it is the only cancer centre on the island and the most important in the country. The option of concentrating cancer experts in this hospital allowed gains of efficiency in consultation by specialised tumour boards, improved treatment and training of young specialists that will strengthen the new oncology units such as in São Vicente Island. The quality and deficiencies of the cancer registry produced by the HAN oncology unit in order to create the hospital-based registry of the same hospital were evaluated in 2014 [4]. At this time, there are no population-based cancer registries on Santiago Island.

Our goal is to be able to be included in the African Cancer Registry Network (AFCRN), fulfilling all the rules of this organisation. In this sense, following a consultancy visit in October 2015, Dr. Goncalo Lacerda (Portuguese expert in Cancer Registries) was invited and supported by the Ministry of Health (MoH) of Cape Verde and the AFCRN to provide 1 week of training to 26 participants coming from different health institutions within Cape Verde [5]. The aim was to train the future registrars to make possible the establishment of hospital-based cancer registries in each central hospital (HAN and Baptista de Sousa in Saint Vincent Island) and a population-based cancer registry under the direction of the MoH and Social Security of Cape Verde.

**Figure 1.**
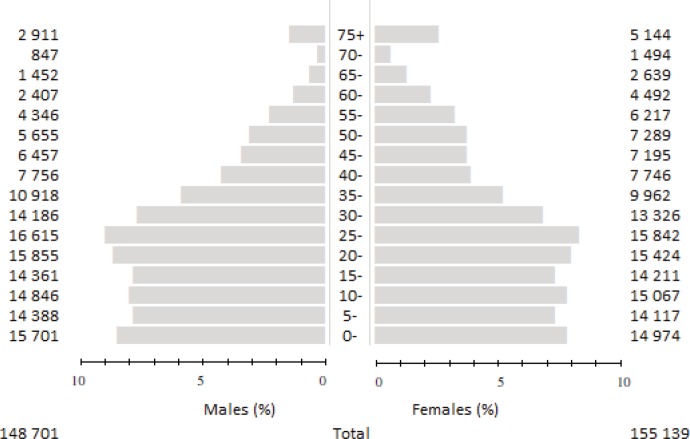
The Santiago Island population pyramid (person-year by sex and age group).

The HAN Cancer Registry established in 2017 is a crucial data source for The Cape Verde Cancer Registry, a population-based cancer registry of the country under construction. The population of Santiago Island is estimated by the National Institute of Statistics of Cape Verde to have been 301,902 inhabitants in 2017/18 (Figure 1) [6].

The objective of this study, promoted by the MoH, was to study Santiago cancer data that is essential to understanding population needs and health system performance, a cornerstone of an effective National Cancer Control Programme in Cape Verde. This study had the support of the Calouste Gulbenkian Foundation and the North Region Cancer Registry of Portugal.

## Methods

This study included cancer patients registered in 2017 and 2018 in the HAN Cancer Registry and dwellers in Santiago Island. The registry collects information from the following sources: medical records, operating room logbooks, ward/admission books, central medical records, histology report books, ultrasonography and computed tomography (CT) reports, specific biochemistry request books, surgical operation lists, nursing report books and follow-up registry. Data were also obtained from the database of the cancer unit of the HAN. Besides, information on the death of cancer patients in the Hospital is also collected. The following variables were extracted: registration number that identifies the patient, sex, age, date of diagnosis, topography and morphology of the tumour (coded according to the 3rd edition of the International Classification of Diseases for Oncology), behaviour, grade and stage [7]. The case finding is entirely active. It is carried out by trained tumour registration officers, who visit the clinical services and collect information using standard registration forms.

However, death certificates were not evaluated. The registry uses CanReg5 software for data entry, management and validation.

### Data analysis

Under the assumption that most cancer cases among Santiago island population were identified at the HAN Cancer Registry, we performed a descriptive analysis of cancer cases diagnosed in 2017–18. Cases with missing or incomplete information, but receiving any type of cancer care in the HAN, were included through the analysis. The results are provided in numbers and proportions.

## Results

In 2017 and 2018, 396 new malignancies were diagnosed on Santiago Island. The most common cancers were the prostate, breast, stomach, cervix and oesophagus, in order of frequency, which together accounted for about half of the oncological pathology of the Santiago Island (56.1% of all cases). As for age distribution, about 90% of cancers were diagnosed at ages equal or superior to 40 years. The highest frequency of cancer was found in individuals aged 75 years or over (21.3%). The histological confirmation rate was 87.8%.

In males, 37.7% of the tumours were of the genitourinary tract and 35.3% of the digestive tract. Prostate cancer was the most frequent cancer. Oesophageal cancer was the second most common type of cancer, followed by the cancer of the stomach and lungs (Table 1).

In females, breast cancer was the most frequent cancer. Cervical cancer was the second most frequent cancer, followed by cancer of the stomach and pancreas (Table 2).

## Discussion

According to the MoH of Cape Verde, most cases were diagnosed at an advanced stage [8]. In 2016 and 2017, 324 and 358 cancer deaths occurred, respectively. The most lethal locations in males were the prostate and the oesophagus, and in females were the breast and the cervix [9]. However, the existing data are somehow fragile [4].

It is necessary and urgent to construct a population-based registry in Cape Verde which allows defining appropriate oncology health strategies. Although the gold standard for cancer registration is population-based cancer registries, these are not feasible in many countries [10, 11]. In these circumstances, a hospital-based cancer registry may provide a starting point for determining cancer occurrence in a region or country [12].

For the first time, we try to determine the burden of cancer at Santiago Island in Cape Verde. HAN is the only hospital with a cancer unit in Santiago Island, and most of the cases are primarily admitted to this hospital. The HAN Cancer Registry is the repository of this essential information.

However, some amount of sub-registry can be suspected since cancer death certificates have not been evaluated and there was no access to information on cancer cases treated outside Cape Verde. Therefore, it is crucial to include cancer data from the death certificate in Cape Verde cancer registry. In future studies, incidence rates should be compared with mortality rates.

This underlines the need for a population-based cancer registry in Cape Verde.

## Conclusions

It is essential to create the conditions for the establishment of Cape Verde’s population-based cancer registry as quickly as possible. Oncological diseases must become obligatory reporting diseases in the country. This report revealed that, in Santiago Island, according to the HAN Cancer Registry, the five most common cancers were the prostate, breast, stomach, cervix and oesophageal in order of frequency.

## Conflicts of interest

There is no conflict of interest.

## Funding

The authors received no financial support for the research, authorship, and/or publication of this article.

## Authors’ contributions

Elizeu Teixeira Silva and Hirondina Borges Spencer acquired data; Elizeu Teixeira Silva, Ana Filipa Gonçalves, Clara Castro, Maria José Bento and Lúcio Lara Santos analysed and interpreted data; Elizeu Teixeira Silva, Lúcio Lara Santos, Ana Filipa Gonçalves, Clara Castro and Maria José Bento drafted the article and critically revised it; Elizeu Teixeira Silva, Hirondina Borges Spencer, Victor Costa, Ana Filipa Gonçalves, Clara Castro, Maria José Bento, Carla Barbosa and Lúcio Lara Santos approved the final version of the draft to be submitted.

## Figures and Tables

**Table 1.** Number of cases by site, age group and percentage: males.

Site	ALL	Age	Age group (years)																%	ICD-10
	age	unk	0-	5-	10-	15-	20-	25-	30-	35-	40-	45-	50-	55-	60-	65-	70-	75+		
Mouth	7	-	-	-	-	-	-	-	2	-	-	-	1	3	-	-	-	1	3,4%	C00-06
Salivary gland	1	-	-	-	-	-	-	-	-	-	-	-	-	1	-	-	-	-	0,5%	C07-08
Nasopharynx	2	-	-	-	-	-	-	1	-	-	1	-	-	-	-	-	-	-	1,0%	C11
Other pharynx	4	-	-	-	-	-	-	-	-	-	1	1	-	1	-	-	-	1	2,0%	C09-10, C12-14
Oesophagus	24	-	-	-	-	-	-	-	-	-	2	3	4	6	4	2	1	2	11,8%	C15
Stomach	22	-	-	-	-	-	-	-	-	-	-	4	4	6	1	2	-	5	10,8%	C16
Colon	6	-	-	-	-	-	-	-	-	-	1	1	1	-	1	1	-	1	2,9%	C18
Rectum	1	-	-	-	-	-	-	-	-	-	-	-	-	-	-	-	1	-	0,5%	C19-20
Anus	1	-	-	-	-	-	-	-	-	-	-	-	-	1	-	-	-	-	0,5%	C21
Liver	11	-	-	-	-	-	-	-	1	1	1	1	2	4	-	-	-	1	5,4%	C22
Gallbladder etc.	-	-	-	-	-	-	-	-	-	-	-	-	-	-	-	-	-	-	-	C23-24
Pancreas	7	-	-	-	-	-	-	-	-	-	-	1	1	1	2	-	-	2	3,4%	C25
Larynx	8	-	-	-	-	-	-	-	-	-	1	3	1	-	2	-	1	-	3,9%	C32
Trachea, bronchus and lung	13	-	-	-	-	-	-	-	-	1	-	3	1	2	1	1	2	2	6,4%	C33-34
Bone	-	-	-	-	-	-	-	-	-	-	-	-	-	-	-	-	-	-	-	C40-41
Melanoma of skin	1	-	-	-	-	-	-	-	-	-	-	-	-	-	-	-	-	1	0,5%	C43
Non-melanoma skin	1	-	-	-	-	-	-	-	-	-	-	-	1	-	-	-	-	-	0,5%	C44
Mesothelioma	-	-	-	-	-	-	-	-	-	-	-	-	-	-	-	-	-	-	-	C45
Kaposi sarcoma	2	-	-	-	-	-	-	-	-	-	-	1	-	-	-	1	-	-	1,0%	C46
Connective and soft tissue	3	-	-	-	-	-	-	-	-	-	1	-	-	1	-	-	-	1	1,5%	C47, C49
Breast	1	-	-	-	-	-	-	-	-	-	-	-	1	-	-	-	-	-	0,5%	C50
Penis	4	-	-	-	-	-	-	-	-	1	-	1	-	-	-	-	1	1	2,0%	C60
Prostate	68	-	-	-	-	-	-	-	-	-	-	3	4	5	6	10	8	32	33,3%	C61
Testis	1	-	1	-	-	-	-	-	-	-	-	-	-	-	-	-	-	-	0,5%	C62
Kidney and renal pelvis	1	-	-	-	-	-	-	-	-	-	-	-	-	1	-	-	-	-	0,5%	C64-65
Bladder	3	-	-	-	-	-	-	-	-	-	-	-	1	1	1	-	-	-	1,5%	C67
Ureter and other urinary	-	-	-	-	-	-	-	-	-	-	-	-	-	-	-	-	-	-	-	C66, C68
Eye	-	-	-	-	-	-	-	-	-	-	-	-	-	-	-	-	-	-	-	C69
Brain and nervous system	1	-	-	-	-	-	-	-	-	-	-	-	-	1	-	-	-	-	0,5%	C70-72
Thyroid	1	-	-	-	-	-	-	-	-	-	-	-	-	-	-	-	1	-	0,5%	C73
Hodgkin lymphoma	1	-	-	-	-	-	-	-	-	-	-	-	-	-	-	-	-	1	0,5%	C81
Non-Hodgkin lymphoma	5	-	1	-	-	-	-	-	1	-	-	-	-	1	1	-	-	1	2,5%	C82-85, C96
Multiple myeloma	-	-	-	-	-	-	-	-	-	-	-	-	-	-	-	-	-	-	-	C90
Lymphoid leukaemia	-	-	-	-	-	-	-	-	-	-	-	-	-	-	-	-	-	-	-	C91
Myeloid leukaemia	-	-	-	-	-	-	-	-	-	-	-	-	-	-	-	-	-	-	-	C92-94
Leukaemia, unspecified	-	-	-	-	-	-	-	-	-	-	-	-	-	-	-	-	-	-	-	C95
Other and unspecified	5	-	-	-	-	-	-	-	-	-	1	1	2	-	-	-	-	1	2,5%	O&U
All sites	205	-	2	-	-	-	-	1	4	3	9	23	24	35	19	17	15	53		C00-96
All sites except C44	204	-	2	-	-	-	-	1	4	3	9	23	23	35	19	17	15	53	100,0%	C00-96 exc. C44

**Table 2.** Number of cases by site age group and percentage: females.

Site	ALL	Age	Age group (years)																%	ICD-10
	age	unk	0-	5-	10-	15-	20-	25-	30-	35-	40-	45-	50-	55-	60-	65-	70-	75+		
Mouth	3	-	-	-	-	-	-	-	-	-	-	1	-	-	1	-	-	1	1,6%	C00-06
Salivary gland	-	-	-	-	-	-	-	-	-	-	-	-	-	-	-	-	-	-	0,0%	C07-08
Nasopharynx	-	-	-	-	-	-	-	-	-	-	-	-	-	-	-	-	-	-	0,0%	C11
Other pharynx	3	-	-	-	-	-	-	-	-	-	-	-	1	2	-	-	-	-	1,6%	C09-10, C12-14
Oesophagus	1	-	-	-	-	-	-	-	-	-	-	-	-	-	1	-	-	-	0,5%	C15
Stomach	19	-	-	-	-	-	-	-	-	-	1	-	2	2	1	-	5	8	9,9%	C16
Colon	3	-	-	-	-	-	-	-	-	-	-	1	1	1	-	-	-	-	1,6%	C18
Rectum	7	-	-	-	-	-	-	-	1	1	-	3	-	-	2	-	-	-	3,6%	C19-20
Anus	-	-	-	-	-	-	-	-	-	-	-	-	-	-	-	-	-	-	0,0%	C21
Liver	3	-	-	-	-	-	-	-	1	-	-	-	-	-	1	-	-	1	1,6%	C22
Gallbladder etc.	3	-	-	-	-	-	-	-	-	-	-	-	-	1	-	-	1	1	1,6%	C23-24
Pancreas	14	-	-	-	-	-	-	-	-	-	-	1	-	2	2	1	2	6	7,3%	C25
Larynx	1	-	-	-	-	-	-	-	-	-	1	-	-	-	-	-	-	-	0,5%	C32
Trachea, bronchus and lung	7	-	-	-	-	-	-	-	-	-	-	1	1	-	-	-	-	5	3,6%	C33-34
Bone	1	-	-	-	-	-	-	-	1	-	-	-	-	-	-	-	-	-	0,5%	C40-41
Melanoma of skin	2	-	-	-	-	-	-	-	-	-	-	-	-	1	-	-	1	-	1,0%	C43
Non-melanoma skin	2	-	-	-	-	-	-	-	-	-	1	-	-	1	-	-	-	-	1,0%	C44
Mesothelioma	-	-	-	-	-	-	-	-	-	-	-	-	-	-	-	-	-	-	0,0%	C45
Kaposi sarcoma	-	-	-	-	-	-	-	-	-	-	-	-	-	-	-	-	-	-	0,0%	C46
Connective and soft tissue	2	-	-	-	-	-	-	-	1	-	-	1	-	-	-	-	-	-	1,0%	C47, C49
Breast	49	-	-	-	-	-	-	-	3	4	9	7	7	6	5	2	1	5	25,5%	C50
Vulva	-	-	-	-	-	-	-	-	-	-	-	-	-	-	-	-	-	-	0,0%	C51
Vagina	-	-	-	-	-	-	-	-	-	-	-	-	-	-	-	-	-	-	0,0%	C52
Cervix uteri	40	-	-	-	-	-	-	1	4	4	4	4	10	5	5	2	-	1	20,8%	C53
Uterus	2	-	-	-	-	-	-	-	-	-	-	-	1	-	-	1	-	-	1,0%	C54-55
Ovary	4	-	-	-	-	1	-	-	-	1	-	-	-	-	-	1	-	1	2,1%	C56
Placenta	-	-	-	-	-	-	-	-	-	-	-	-	-	-	-	-	-	-	0,0%	C58
Kidney and renal pelvis	2	-	-	-	-	-	-	-	-	-	-	-	-	1	1	-	-	-	1,0%	C64-65
Bladder	-	-	-	-	-	-	-	-	-	-	-	-	-	-	-	-	-	-	0,0%	C67
Ureter and other urinary	-	-	-	-	-	-	-	-	-	-	-	-	-	-	-	-	-	-	0,0%	C66, C68
Eye	1	-	-	-	-	-	-	-	-	-	-	-	1	-	-	-	-	-	0,5%	C69
Brain and nervous system	4	-	-	1	-	1	1	-	-	-	-	-	-	-	-	1	-	-	2,1%	C70-72
Thyroid	7	-	-	-	-	-	1	-	-	-	2	1	2	-	-	-	-	1	3,6%	C73
Hodgkin lymphoma	-	-	-	-	-	-	-	-	-	-	-	-	-	-	-	-	-	-	0,0%	C81
Non-Hodgkin lymphoma	6	-	2	-	-	-	-	-	-	-	-	-	-	3	1	-	-	-	3,1%	C82-85, C96
Multiple myeloma	1	-	-	-	-	-	-	-	-	-	-	-	-	-	1	-	-	-	0,5%	C90
Lymphoid leukaemia	2	-	-	-	-	-	-	-	-	-	-	-	-	-	-	-	-	2	1,0%	C91
Myeloid leukaemia	2	-	-	-	-	-	-	-	-	-	-	1	-	-	1	-	-	-	1,0%	C92-94
Leukaemia, unspecified	1	-	-	-	-	-	-	-	-	-	-	-	-	-	1	-	-	-	0,5%	C95
Other and unspecified	2	-	-	-	-	-	-	1	-	-	-	-	1	-	-	-	-	-	1,0%	O&U
All sites	194	-	2	1	-	2	2	2	11	10	18	21	27	25	23	8	10	32		C00-96
All sites except C44	192	-	2	1	-	2	2	2	11	10	17	21	27	24	23	8	10	32	100,0%	C00-96 exc. C44
